# An Analysis Based on Molecular Docking and Molecular Dynamics Simulation Study of Bromelain as Anti-SARS-CoV-2 Variants

**DOI:** 10.3389/fphar.2021.717757

**Published:** 2021-08-20

**Authors:** Trina Ekawati Tallei, Fatimawali, Afriza Yelnetty, Rinaldi Idroes, Diah Kusumawaty, Talha Bin Emran, Talha Zahid Yesiloglu, Wolfgang Sippl, Shafi Mahmud, Taha Alqahtani, Ali M. Alqahtani, Saeed Asiri, Mohammed Rahmatullah, Rownak Jahan, Md. Arif Khan, Ismail Celik

**Affiliations:** ^1^ Department of Biology, Faculty of Mathematics and Natural Sciences, Sam Ratulangi University, Manado, Indonesia; ^2^ The University Centre of Excellence for Biotechnology and Conservation of Wallacea, Institute for Research and Community Services, Sam Ratulangi University, Manado, Indonesia; ^3^ Pharmacy Study Program, Faculty of Mathematics and Natural Sciences, Sam Ratulangi University, Manado, Indonesia; ^4^ Department of Animal Production, Faculty of Animal Husbandry, Sam Ratulangi University, Manado, Indonesia; ^5^ Department of Pharmacy, Faculty of Mathematics and Natural Sciences, Universitas Syiah Kuala, Banda Aceh, Indonesia; ^6^ Department of Biology, Faculty of Mathematics and Natural Sciences Education, Universitas Pendidikan Indonesia, Bandung, Indonesia; ^7^ Department of Pharmacy, BGC Trust University Bangladesh, Chittagong, Bangladesh; ^8^ Institute of Pharmacy, Martin-Luther University of Halle-Wittenberg, Halle, Germany; ^9^ Microbiology Laboratory, Department of Genetic Engineering and Biotechnology, University of Rajshahi, Rajshahi, Bangladesh; ^10^ Department of Pharmacology, College of Pharmacy, King Khalid University, Abha, Saudi Arabia; ^11^ Department of Clinical Laboratory Sciences, College of Applied Medical Sciences, Najran University, Najran, Saudi Arabia; ^12^ Department of Biotechnology and Genetic Engineering, University of Development Alternative, Dhaka, Bangladesh; ^13^ Department of Pharmaceutical Chemistry, Faculty of Pharmacy, Erciyes University, Kayseri, Turkey

**Keywords:** bromelain, receptor-binding domain, SARS-CoV-2, mutation, variants

## Abstract

The rapid spread of a novel coronavirus known as SARS-CoV-2 has compelled the entire world to seek ways to weaken this virus, prevent its spread and also eliminate it. However, no drug has been approved to treat COVID-19. Furthermore, the receptor-binding domain (RBD) on this viral spike protein, as well as several other important parts of this virus, have recently undergone mutations, resulting in new virus variants. While no treatment is currently available, a naturally derived molecule with known antiviral properties could be used as a potential treatment. Bromelain is an enzyme found in the fruit and stem of pineapples. This substance has been shown to have a broad antiviral activity. In this article, we analyse the ability of bromelain to counteract various variants of the SARS-CoV-2 by targeting bromelain binding on the side of this viral interaction with human angiotensin-converting enzyme 2 (hACE2) using molecular docking and molecular dynamics simulation approaches. We have succeeded in making three-dimensional configurations of various RBD variants using protein modelling. Bromelain exhibited good binding affinity toward various variants of RBDs and binds right at the binding site between RBDs and hACE2. This result is also presented in the modelling between Bromelain, RBD, and hACE2. The molecular dynamics (MD) simulations study revealed significant stability of the bromelain and RBD proteins separately up to 100 ns with an RMSD value of 2 Å. Furthermore, despite increases in RMSD and changes in Rog values of complexes, which are likely due to some destabilized interactions between bromelain and RBD proteins, two proteins in each complex remained bonded, and the site where the two proteins bind remained unchanged. This finding indicated that bromelain could have an inhibitory effect on different SARS-CoV-2 variants, paving the way for a new SARS-CoV-2 inhibitor drug. However, more *in vitro* and *in vivo* research on this potential mechanism of action is required.

## Introduction

Nature has an abundance of biological compounds that can be used as drug candidates to treat a wide range of diseases, both infectious and degenerative. Today, research is focused on discovering therapeutic agents that are derived from natural ingredients, such as plant materials. Pineapple, where the fruit is frequently consumed by the community, is one of the plants with nutraceutical properties. This plant has been used as a medicinal agent for a long time, and some of its benefits have been scientifically established. Bromelain, the key component of pineapple fruit and roots, is one of many active compounds found in pineapple that possesses therapeutic properties. Bromelain is made up of a variety of proteases, as well as phosphatase, glucosidase, peroxidase, cellulases, and glycoprotein (Bhattacharyya, 2008). Minor thiol endopeptidase, ananain, comosain, protease inhibitors, and organically bound calcium are all present in pineapple bromelain (Gautam et al., 2010; Bala et al., 2013).

Fresh pineapple is one of the favorite fruits in tropical countries. Apart from its nutritional value, bromelain’s active ingredients have been shown to have therapeutic properties, such as antiviral (e.g. anti-SARS-CoV-2), anti-inflammatory, anticancer, antimicrobial, and immunomodulatory function (Dopheide and Ward, 1981; Chandler and Mynott, 1998; Rathnavelu et al., 2016; Sagar et al., 2020; Chakraborty et al., 2021). Bromelain has a half-life of 6–9 h (Castell et al., 1997) and a plasma concentration of 2.5–4 ng/ml after an oral dose of 8.6 g per day (Kumakura et al., 1988). In an artificial stomach juice, the bromelain concentration of 3.66 mg/ml was detected after 4 h and it remained in artificial blood at a concentration of 2.44 mg/ml after 4 h (Pavan et al., 2012). According to recent research, bromelain can inhibit SARS-CoV-2 infection in VeroE6 cells by lowering the expression of the host cell receptor angiotensin-converting enzyme 2 (ACE-2) and the primed SARS-CoV-2 spike (S) protein. Bromelain has thus been suggested as an antiviral agent for COVID-19 therapy (Sagar et al., 2020). In another study, bromelain at a concentration of 100 g/ml was found to be effective in dissolving SARS-COV-2 spikes and envelope proteins. When combined with 20 mg/ml of acetylcysteine, the SARS-COV-2 spike and envelope proteins are fully disintegrated (Akhter et al., 2020). Multiple SARS-CoV-2 variants have recently been reported as circulating globally, according to the Center for Disease Control and Prevention (CDC). To date, at least four variants have been identified, the most notable of which are the B.1.1.7 lineage (the United Kingdom/United Kingdom variant/Alpha variant), B.1.351 lineage (South Africa/SA variant/Beta variant), P.1 lineage (Brazil/BR variant/Gamma variant), and B.1.429 lineage (California/US variant/Epsilon variant). The B.1.1.7, B.1.351, and P.1 lineages are considered variants of concern (VOC) because they have demonstrated a clear impact on disease transmission and severity, as well as immunity that may influence disease epidemiological situations. Meanwhile, the B.1.429 lineage is classified as a variant of interest because there is evidence that this variant possesses a mutation that alters the mode of transmission, the sensitivity of the test kit, the severity of symptoms, and the virus’s ability to evade the immune system. However, there is currently insufficient evidence, necessitating additional research.

The B.1.1.7 lineage has a mutation in the spike glycoprotein’s receptor-binding domain (RBD) at position 501, where the amino acid asparagine (N) has been replaced with tyrosine (Y) (abbreviated as N50Y). The B.1.351 lineage has multiple mutations in the spike protein which include K417N, E484K, and N501Y. The P.1 lineage has three RBD spike mutations: K417T, E484K, and N501Y. The B.1.429 lineage has the L452R mutation. Thus, these variants have mutations in the spike glycoprotein Xie et al. (2021), where this protein is responsible for the entry of the virus into the host cell through its attachment to the angiotensin-converting enzyme 2 (ACE2) receptor (Ni et al., 2020; Shang et al., 2020). A structural study analysis revealed that the RBD of the SARS-CoV-2 spike glycoprotein contains residues that are required for ACE2 binding (Lan et al., 2020). This receptor is a type I membrane protein (single transmembrane) protein found in several organs, including the oral and nasal mucosa, nasopharynx, lung, arteries, heart, kidneys, stomach, and intestines (Gheblawi et al., 2020; Bian & Li, 2021). As a result, several researchers have proposed spike protein as one of the targets for anti-SARS-CoV-2 drugs (Huang et al., 2020; Prajapat et al., 2020; Tallei et al., 2020; Khairan et al., 2021).

In the management of coronavirus disease 2019 (COVID-19), patients are treated with antiviral drugs (remdesivir, lopinavir, umifenovir, favipiravir, and oseltamivir), viral protease inhibitors (lopinavir and darunavir), anti-inflammatory agents (tocilizumab), and antimalarials (chloroquine and hydroxychloroquine) (Singh et al., 2020; Wu et al., 2020). However, there are still concerns about the efficacy of these drugs due to a lack of valid clinical trial data. As a result, natural compounds remain a promising alternative. Although bromelain has been shown to inhibit SARS-CoV-2 replication *in vitro*, with the development of new variants of this virus, computer modelling using molecular docking approaches and molecular dynamics simulation is required to evaluate bromelain’s ability to inhibit various variants of this virus. The purpose of this study was to investigate the interaction between bromelain and various mutated SARS-CoV-2 receptor-binding domains.

## Materials and Methods

### Preparation of Fruit Bromelain 3D Structure

The crystal structure of fruit bromelain (Bro) was unavailable in the PDB data-bank, thus homology modelling was utilized to generate its 3D structure using SWISS-MODEL (Schwede et al., 2003). The SWISS-MODEL structure assessment online server was used for validation (https://swissmodel.expasy.org/assess/) (Waterhouse et al., 2018). The protein sequence of fruit bromelain was obtained from GenBank with accession number QIM61761.1 (https://www.ncbi.nlm.nih.gov/protein/QIM61761.1).

### Receptor Preparation

The crystal structure of the SARS-CoV-2 spike receptor-binding domain (RBD) was retrieved from the Protein Data Bank with PDB ID 6YLA (https://www.rcsb.org/structure/6YLA), assigned as wild-type (WT). The 3D structure WT and its variants were created using homology modelling on the SWISS Model web-server and assigned as BR (P.1 lineage), SA (B.1.351 lineage), United Kingdom (B.1.1.7 lineage), and United States (B.1.427 lineage). Energy minimization was employed to overcome minor structural distortions, unfavorable interactions, and collisions introduced during the modelling phase.

### Multiple Alignment Analysis

The multiple alignment analysis of the amino acid sequences of RBD WT and its variants was performed using the UCSF Chimera package release 1.15 (Pettersen et al., 2004).

### Molecular Docking Analysis

The simulation of molecular docking was performed on the ClusPro protein-protein docking server (https://cluspro.bu.edu) (Kozakov et al., 2013; Kozakov et al., 2017; Vajda et al., 2017; Desta et al., 2020). The docking results were visualized in the LigPlot to confirm the binding position of bromelain and the receptors. As a comparison, other interaction graphics were generated using the EMBL-EBI tool PDBsum (http://www.ebi.ac.uk/thornton-srv/databases/pdbsum/Generate.html). The binding affinity (ΔG) and dissociation constant (KD) predicted values were obtained from the Prodigy server (https://wenmr.science.uu.nl/prodigy/) (Vangone & Bonvin, 2015; Xue et al., 2016). The UCSF Chimera package release 1.15 was used to display the position of the interaction between RBD, hACE2, and bromelain.

### Binding Free Energy Calculation of the Complexes

Estimations of the binding energies for complexes of Bro with RBD of wild type, B.1.427, B.1.1.7, B.1.351, and P.1 lineages were performed by using the MM-GBSA method. The method is a combination of molecular mechanics approaches with the generalized Born method and the surface area continuum solvation model.

$$\Delta {\text{G}}_{binding\;free\;energy}=G_{\begin{array}{c} protein+ligand\\ complex \end{array}}-\left(G_{protein}+\;G_{ligand}\right)$$ (1)

Here, each energy in Eq. 1 is a sum of vdW, internal, electrostatic, GB, and SA energy terms as shown in Eq. 2.

$$\text{G}={\text{E}}_{int}+{\text{E}}_{ele}+{\text{E}}_{vdw}+{\text{G}}_{GB}+{\text{G}}_{SA}$$ (2)

Eint (internal) refers to bond, angle, and dihedral energies and Eele (electrostatic) refers to Coulomb force, while Evdw (van der Waals) refers to van der Waals interactions values. In the equation, GGB is the electrostatic solvation energy and, lastly, GSA is the non-electrostatic solvation energy. The MMGBSA. py (Hou et al., 2011; Miller et al., 2012) command which is implemented in Amber18 software was used to calculate energy values.

### Molecular Dynamics (MD) Simulation

The dynamic behavior of Bro with WT, B.1.1.7, B.1.351, P.1, and B.1.427 lineage complexes was studied by MD simulation performed on Amber18 (Case et al., 2018) using the FF14SB protein force field. The solvation of the system was done by using the TIP3P water model with a margin of 10 Å (Jorgensen et al., 1983; Maier et al., 2015). Following that, the system was neutralized by the addition of counter ions. Thenceforward, two consecutive minimization steps were performed before the long MD simulation. In the first, 3,000 iterations (1,000 steepest descent and following 2,000 conjugate gradient) were submitted. In the second minimization step, 4,000 iterations (2,000 steepest descent and following 2,000 conjugate gradient) were performed. In the first minimization step, atom coordinates for the whole system were restrained to their initial coordinates with a force constant of 10 kcal/mol Å^−2^. In the second minimization step, the whole system was freely minimized to relieve atomic clashes/contacts in the entire system. The system was then heated at 300 K through 100 ps of MD with a time step of 2 fs per step while the whole system was restrained again with a force constant of 10 kcal mol-1 Å-2. That was followed by density evaluation of the system through 100 ps of MD with a time step of 2 fs per step. Before the long MD simulation, an equilibration step (a short MD for 200 ps) was carried out to equilibrate the system. Finally, a routine MD simulation for 100 ns was applied during which the temperature was kept at 300 K. The LEaP module of AMBER was used for protein-protein complex preparation, counter ion addition, solvation, and preparation of topology files. The PMEMD. CUDA GPU implementation was used for MD simulation production and the CPPTRAJ package of Amber18 was used for trajectory processing (Salomon-Ferrer et al., 2013).

## Results and Discussion

The mutated variant of SARS-CoV-2 has spread worldwide and raised concerns about the vaccine’s effectiveness. The B.1.1.7 lineage is more difficult to neutralize than the parental virus, thus weakening the neutralization process by some members of a major class of public antibodies through light-chain interacting with the residue 501. This means that the virus can change the immune domains in a variety of ways to escape human immunity while still infecting and causing disease (Supasa et al., 2021).

The B.1.1.7 lineage was said to have started in the United Kingdom (Challen et al., 2021). The deletions at 69–70, 144, and substitutions K417N, K417T, E484K, N501Y, A570D, D614G, P681H, T716I, S982A, D1118H, and many others in the spike protein are predicted to affect SARS-CoV-2’s ability to transmit and infect. The N501Y is located on the ACE2 interacting surface (Supasa et al., 2021). This could have the consequence that the currently developed vaccine will not be effective against this B.1.1.7 lineage (Harrington et al., 2021). The variants BR and SA have also been reported to be more contagious. Many researchers around the world are thus using computational methods and techniques to identify new nature-based agents to solve this problem and reduce the fear of a pandemic.

Based on the information above, we considered natural resource-based management options for COVID-19. Bromelain, the active compound contained in pineapple fruit and stem, was discovered to have the ability to disintegrate spike protein in recent research (Sagar et al., 2020), and also alter the viral protein (Akhter et al., 2020). The interaction between Bro and RBDs is discussed in this article.

### Fruit Bromelain 3D Structure

**FIGURE 1:**
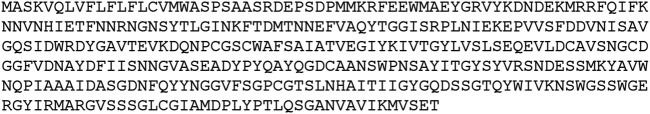
The amino acid sequence of fruit bromelain of pineapple (*Ananas comosus*) retrieved from GenBank with accession number QIM61761.1.

GenBank provides information about proteins that have been published in previous research. The platform provides information, among others, about catalytic activity residues. Our study utilizes this platform to obtain information on protein sequences from the fruit bromelain (*Ananas comosus* (L.) Merr.), which we then use to do homology modelling. The sequence of Bro extracted from pineapple fruit obtained from GenBank is shown in Figure 1.

**FIGURE 2:**
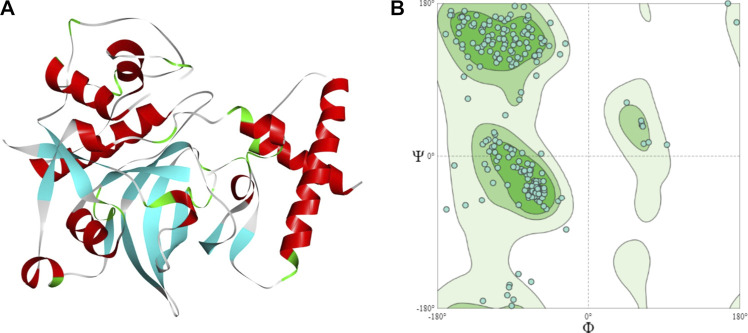
**(A)** The 3D structural model of bromelain fruit prepared using homology modelling **(B)** Ramachandran plot showing the residues in the most favoured regions of 90.55%.

The sequence has a cathepsin pro-peptide inhibitor domain (inhibitor 129 superfamily) at the N-terminus and a papain family cysteine protease (peptidase C1 superfamily) at the other end. The 3D structure of the fruit bromelain prepared using homology modelling on the SWISS-MODEL web-server is displayed in Figure 2A. A good model will typically have 90% of its residues in the Ramachandran plot’s allowable regions (Laskowski et al., 1993), and this information is in accordance with the model generated by SWISS-MODEL structure assessment (Figure 2B).

### Multiple Alignment Analysis

**FIGURE 3:**
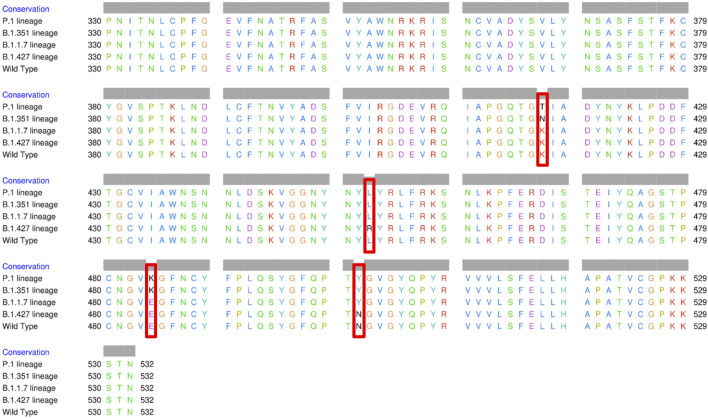
Multiple sequence alignment of the amino acid sequences of wild type (WT) SARS-CoV-2 spike receptor-binding domain (RBDs) and the four variants BR, SA, United Kingdom, and US.

The spike glycoprotein comprises of the S1 subunit (14–685 residues), which is responsible for receptor binding, and the S2 subunit (686–1,273 residues), which is responsible for membrane fusion. The RBD is located at 319–541 residues (Huang et al., 2020). In this report, we used the S1 subunit of spike protein, where there is a receptor-binding domain (RBD), which is responsible for the attachment of the virus to hACE2. The RBD amino acid sequences of all variants analyzed in this study were aligned using chimera release 1.15 (Figure 3). The locations of all mutations were indicated. The WT has no mutations, the P.1 lineage (BR) has mutations at K417T, E484K, and N501Y, the B.1.351 lineage (SA) has mutations at K417N, E484K, and N501Y, the B.1.1.7 lineage (UK) has only one mutation at N501Y (Davies et al., 2021; Khan et al., 2021; Madhi et al., 2021; Wang et al., 2021), and the B.1.427 lineage (US) carries L452R mutation (Deng et al., 2021). Khan et al. (2021) further suggested that the N501Y-E484K variants hypothetically alter the binding affinity of RBD to hACE2, establish new interprotein contacts, and change the internal structural dynamics, resulting in increased binding and infectivity. The L452R mutation circumvents human leukocyte antigen (HLA)-restricted cellular immunity and enhances binding affinity for the viral receptor hACE2 (Motozono et al., 2021). The SA and BR mutations are significantly more lethal than the United Kingdom variant. However, it is reported that each of these mutants changes the binding affinity, establishes new inter-protein contacts, and modifies the internal structural dynamics, thereby increasing binding and eventually infectivity (Khan et al., 2021).

### Molecular Docking Analysis

Protein-protein docking is a molecular modelling technique that aims to predict the mutual orientation and position of two molecules forming a complex using computer algorithms and techniques. Simulation of molecular docking was performed on the Cluspro web-server (https://cluspro.bu.edu/login.php) to determine the possibility of interaction between RBDs and Bro. Docking is a method of interacting between molecules to determine the lowest energy created when a stable complex forms as a result of docking events. ClusPro ranks docking models based on the size of the conformation cluster to which they belong. ClusPro also provides two types of docking energies: the lowest energy among the conformations within a cluster of conformations and the core energy of the cluster (Xue et al., 2011).

The ClusPro score reflects the attempt to find the native site with the lowest free binding energy. Table 1 shows the size (number of members) of each cluster, the weighted energy score of the cluster center (i.e., the structure with the most neighboring structures in the cluster), and the energy score of the cluster’s lowest energy structure (Kozakov et al., 2017). As a result of mutations in RBD, the ClusPro score increased from −817.2 to −844.3 kJ/mol in Bro-UK variant complex and decreased to −744.3, −744.5, and −636.0 kJ/mol, in the Bro-BR, SA, and US variant complexes, respectively. It appears that Bro has a higher preference for the RBD United Kingdom variant compared to other RBDs. However, according to Kozakov et al. (2017), the ClusPro score should not be regarded as a measure of binding affinity. The native fold was typically the cluster with the largest number of low-energy structures (Comeau et al., 2004). The prodigy results for the ΔG calculation indicated that Bro binds to the BR and SA variants slightly stronger. The dissociation constant (K_D_) represents the equilibrium constant that exists when molecules bonded together in a complex dissociate. The smaller the dissociation constant value, the more tightly bonded a molecular complex is, or the greater the affinity between the molecules in the complex. Inferring from the binding affinity and dissociation constant values, Bro binds slightly more strongly to RBD variants than to WT.

**TABLE 1:** Weighted scores, binding affinity (ΔG), and dissociation constant (Kd) of the interaction of each RBD with bromelain.

RBD	Cluster	Members (docked conformations)	Representative	Weighted score KJ/mol	ΔG (kcal mol)	Kd (M) at 25.0°C
WT	0	70	Center	−717.5	−14.9	1.1^–11^
			Lowest energy structure	−819.3	—	—
United Kingdom	0	67	Center	−834.2	−15.0	9.2^–12^
			Lowest energy structure	−844.3	—	—
BR	0	80	Center	−744.3	−15.6	3.7^–12^
			Lowest energy structure	−744.3	—	—
SA	0	92	Center	−680.0	−15.4	5.1^–12^
			Lowest energy structure	−744.5	—	—
United States	1	114	Center	−534.9	−15.0	9.2^–12^
			Lowest energy structure	−636.0	—	—

**FIGURE 4:**
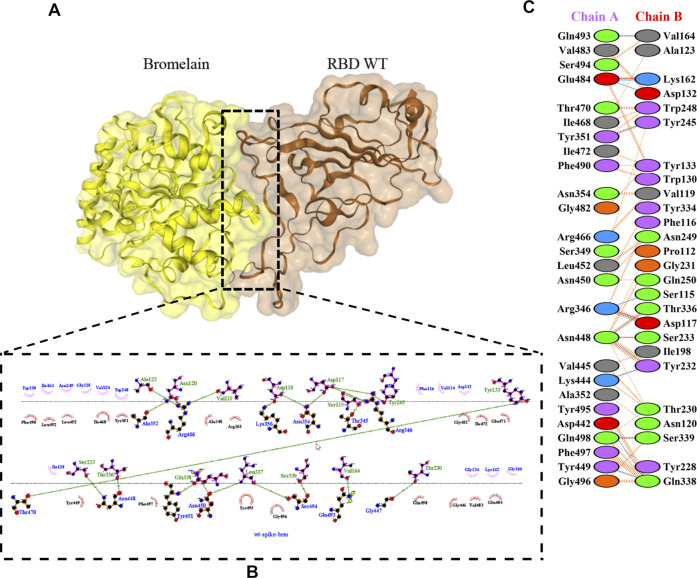
Docking representation of the WT and bromelain complex. **(A)** the binding interface of the complex, **(B)** the binding interaction between the amino acids, and **(C)** interaction representation including hydrogen, salt bridges, and nonbonded interactions. Chain A is RBD WT and chain B is bromelain.

**FIGURE 5:**
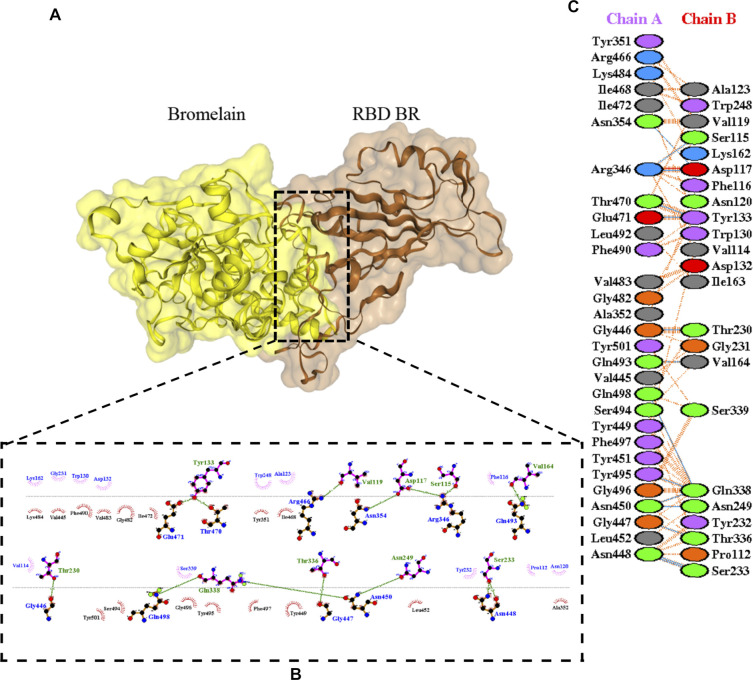
Docking representation of the BR variant and bromelain complex. **(A)** the binding interface of the complex, **(B)** the binding interaction between the amino acids, and **(C)** interaction representation including hydrogen, salt bridges, and nonbonded interactions. Chain A is RBD BR and chain B is bromelain.

**FIGURE 6:**
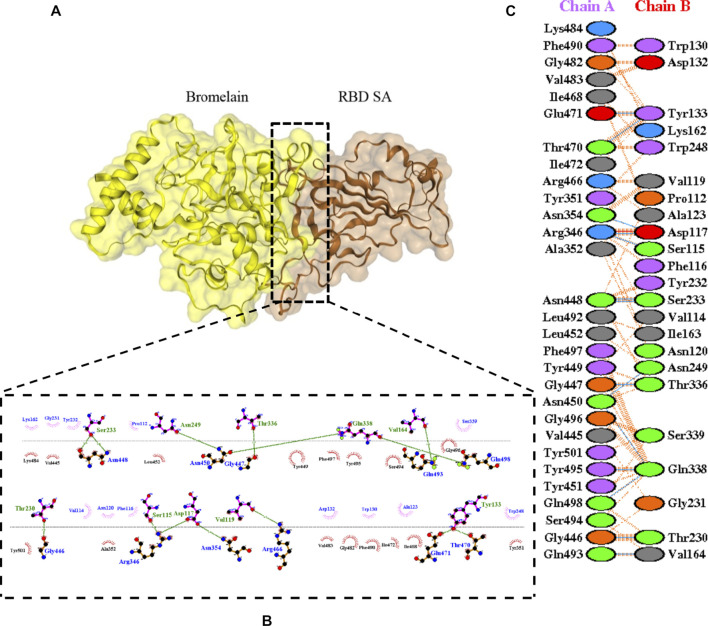
Docking representation of the SA variant and bromelain complex. **(A)** the binding interface of the complex, **(B)** the binding interaction between the amino acids, and **(C)** interaction representation including hydrogen, salt bridges, and nonbonded interactions. Chain A is RBD SA and chain B is bromelain.

**FIGURE 7:**
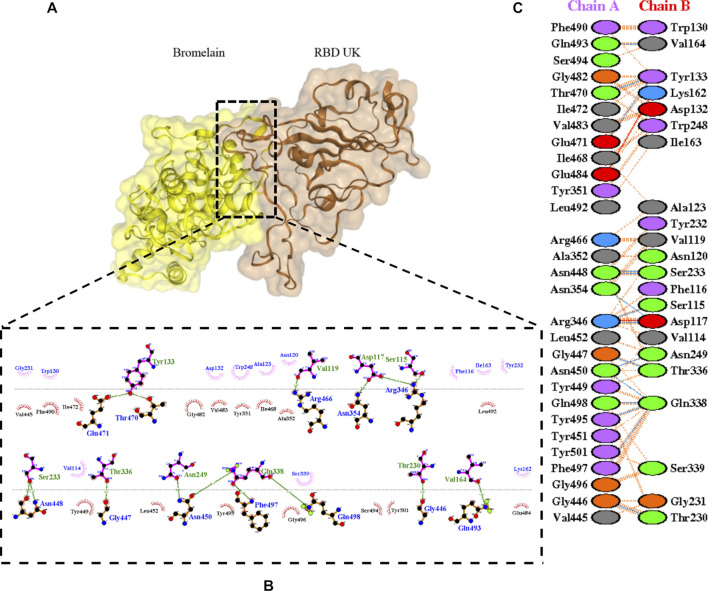
Docking representation of the United Kingdom variant and bromelain complex. **(A)** the binding interface of the complex, **(B)** the binding interaction between the amino acids, and **(C)** interaction representation including hydrogen, salt bridges, and nonbonded interactions. Chain A is RBD United Kingdom and chain B is bromelain.

**FIGURE 8:**
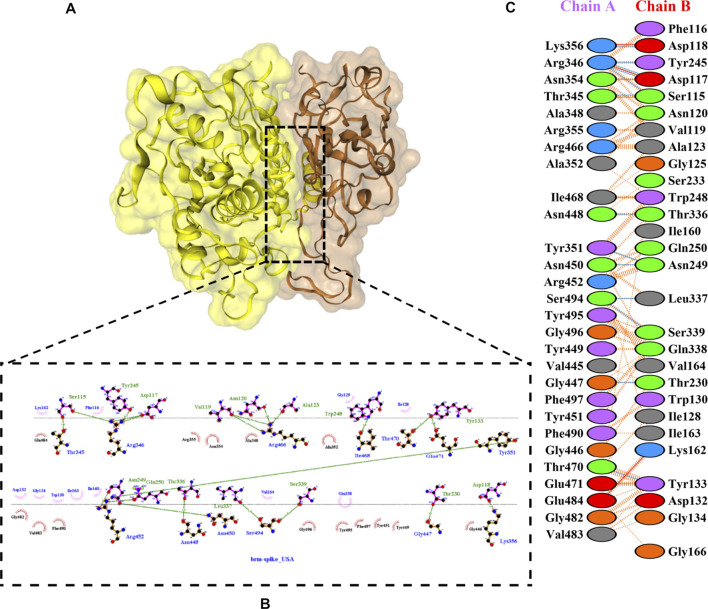
Docking representation of the United States variant and bromelain complex. **(A)** the binding interface of the complex, **(B)** the binding interaction between the amino acids, and **(C)** interaction representation including hydrogen, salt bridges, and nonbonded interactions. Chain A is RBD United States and chain B is bromelain.

The molecular docking complexes generated by ClusPro were used to analyze the amino acid residue interaction between Bro and RBDs. The interaction was generated by the EMBL-EBI tool PDBsum and LigPlot+ (Figure 4–8). Cannalire et al. (2020) reported that the RBD of SARS-CoV Tyr442, Leu472, Asn479, Thr487, and Tyr491 to bind to ACE2. On the other hand, several studies have shown that the key amino acids from the RBD of SARS-CoV-2, Leu455, Phe486, Gln493, and Asn501 form stronger interactions with the host receptor, together with Lys417, thus causing SARS-CoV-2’s affinity for ACE2 to be 20 folds compared to SARS-CoV (Lan et al., 2020; Yan et al., 2020). According to Koley et al. (2021), the interacting residues of RBD are Gln493, Tyr449, and Gly446. Khan et al. (2021), on the other hand, reported that hACE2-RBD forms hydrogen bonds between Glu30-Lys417, Glu35-Gln493, Glu38-Tyr449, Glu38-Gly496, Tyr41-Thr500, Tyr41-Thr500, Gln42-Gln498, Asn330-Thr500, Lys353-Gly502, Lys353-Gly496, and Lys353-Gln498, and also a salt bridge between amino acids Glu30 and Lys417.

Table 2 shows the interacting residues between Bro and RBDs, where Bro occupies the site where hACE2 binds to RBD. Bro binds with Tyr449, Glu484, Gln493, Gly496, and Gln498 in RBD-WT, with Tyr449, Gly446, Glu484, Gln493, Gly496, Gln498, and Tyr501 in RBD-UK, with Tyr449, Gly446, Lys484, Gln493, Gly496, Gln498, and Tyr501 in RBD-BR, with Tyr449, Gly446, Lys484, Gln493, Gly496, Gln498, and Tyr501 in RBD-SA, and with Tyr449, Gly446, Arg452, Glu484, and Gly496 in RBD-US. This suggests that bromelain binds more to BRD variants than WT, except with the US variant. The substitution sites E484K (Glu484 → Lys484) and N501Y (Asn501 → Tyr501) in RBD variants also have bonds and nonbonded contacts with Bro. Bro has several nonbonded contacts with Arg452 in the US variant in the substitution site (L452R). Bro does not have interaction in the substitution site K417T (Lys417 → Thr417).

**TABLE 2:** The position of the interacting residues of bromelain with RBDs pocket and mutation sites (highlighted in bold). Key amino acid residues that play a role in binding RBD to hACE2 are marked with an italic font.

RBD variants	RBD	Bromelain	Distance (Å)		
	Residues	Residues	H-bonds	Salt bridge	Non-bonded contacts
WT	*Tyr449*	Gln338	3.05	—	3.22; 3.28; 3.05
	Glu484	Lys162	2.50	2.50	2.89; 3.71; 2.79; 3.79; 2.50
		Asp132	2.93	—	2.93
		Tyr133	—	—	3.63; 3.69; 3.58; 2.89; 3.28
	*Gln493*	Val164	2.89	—	3.75; 3.80; 2.89
	*Gly496*	Gln338	—	—	3.84; 3.28; 3.44; 3.61; 3.29; 3.74
		Ser339	—	—	3.40; 3.89; 3.81
	Gln498	Gln338	—	—	3.59; 3.87; 3.65
	—	Ser339	2.96	—	3.61; 3.17; 3.59; 3.72; 2.96
	—	Tyr228	—	—	3.85
	—	Tyr230	—	—	3.61
	—		—	—	
United Kingdom	*Tyr449*	Thr336	—	—	3.81; 3.78
		Gln338	—	—	3.16; 3.30; 3.89; 3.57
		Val114	—	—	3.85
	Gly446	Thr230	2.75	—	3.81; 3.21; 3.84; 3.72; 3.67; 3.25; 3.48; 2.95; 2.75
		Gly231	—	—	3,45
	Glu484	Asp132	—	—	3.83
		Lys162	—	2.58	2.79; 3.36; 2.58; 2.59
	*Gln493*	Val164	2.89	—	3.65; 3.76; 2.89
	*Gly496*	Gln338	—	—	3.54; 3.76; 3.25; 3.23; 3.89; 3.17; 2.78
		Ser339	—	—	3.43; 3.88; 3.65; 3.56; 3.78; 3.26
	*Gln498*	Gln338	2.81	—	3.58; 3.65; 2.81
		Ser339	—	—	3.59; 3.67
		Th2230	—	—	3.44; 3.68; 2.81
	*Tyr501*	Ser339	3.48	—	—
BR	*Tyr449*	Thr336	—	—	3.79; 3.78
		Gln338	—	—	3.14; 3.28; 3.81; 3.61
		Val114	—	—	3.87
	Gly446	Thr230	2.76	—	3.82; 3.21; 3.69; 3.65; 3.21; 3.44; 2.95; 2.76; 3.42
	Lys484	Lys162	—	—	3.81, 3.72
	*Gln493*	Val164	2.86	—	3.42; 3.61; 2.86
	*Gly496*	Gln338	—	—	3.51; 3.86; 3.81; 3.27; 3.18; 3.21; 2.76
		Ser339	—	—	3.44; 3.85; 3.71; 3.63; 3.81; 3.28
	*Gln498*	Gln338	2.81	—	3.63; 3.67; 2.81
		Ser339	—	—	3.61; 3.69
		Thr230	—	—	3.42; 3.63; 2.81
	*Tyr501*	Ser339	3.52	—	—
SA	*Tyr449*	Thr336	—	—	3.79; 3.77
		Gln338	—	—	3.14; 3.28; 3.81; 3.61
		Val441	—	—	3.90
	Gly446	Thr230	2.76	—	3.82; 3.22; 3.83; 3.69; 3.65; 3.20; 3.43; 2.95; 2.76
		Gly231	—	—	3.41
	Lys484	Lys162	—	—	3.82; 3.73
	*Gln493*	Val164	2.88	—	3.44; 3.65; 2.88
	*Gly496*	Gln338	—	—	3.51; 3.86; 3.81; 3.27; 3.17; 3.21; 2.76
		Ser339	—	—	3.44; 3.87; 3.71; 3.63; 3.81; 3.27
	*Gln498*	Gln338	2.81	—	3.64; 3.67; 2.81
		Ser339	—	—	3.61; 3.69
		Thr230	—	—	3.63; 3.57; 2.81
	*Tyr501*	Ser339	3.52	—	—
US	*Tyr449*	Gln338	—	—	2.99; 3.27; 3.54
	Gly446	Thr230	—	—	3.43; 3.28
	Arg452	Asn249	—	—	3.72; 3.74; 3.12; 3.75; 2.68; 3.39; 2.89; 3.68; 3.23
		Gln250	—	—	3.71; 3.67; 2.62
		Ile163	—	—	3.66
		Ile160	—	—	3.28
	Glu484	Tyr133	—	—	3.78
		Lys162	—	2.59	3.83; 3.56; 2.92; 3.61; 2.80; 3.38; 2.59
		Gly166	—		3.78
	*Gly496*	Gln338	—		3.27; 3.65
		Ser339	—		3.75

**FIGURE 9:**
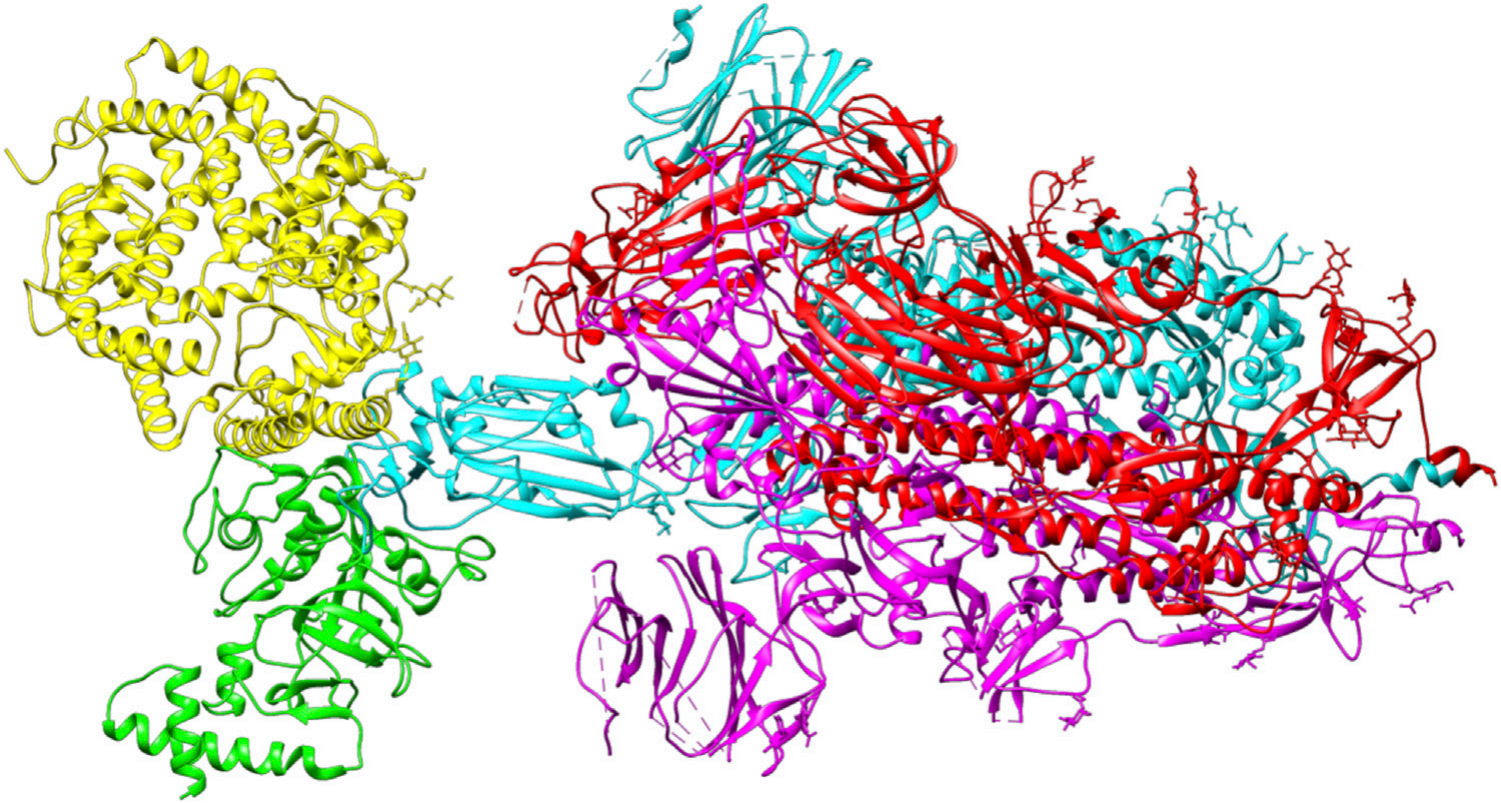
The competitive binding model between bromelain (green) and the ACE2 receptor (yellow) on the binding site of RBD (light blue) of the spike glycoprotein.

ACE2 and Bro can be considered competitive because they do not show a complete allosteric binding. It may be connected right next to the binding site of RBD and prevent the virus from binding with ACE2. Figure 9 illustrates the competitive binding model between bromelain and the ACE2 receptor at the RBD binding site. Several previous studies have shown that Bro is capable of removing the spike protein from a variety of viruses (Kennedy, 1974; Kharitonenkov et al., 1978). Bro has also been shown to be capable of inhibiting SARS-CoV-2 infection in VeroE6 cells via a disulfide bond-mediated mechanism (Sagar et al., 2020). Due to the paucity of research on the inhibition of SARS-CoV-2 RBD by plant-based proteins/enzymes, comparative data is difficult to obtain. However, considerable research has been conducted on the inhibitory effect of peptides on RBD (Barh et al., 2020; Salman et al., 2020; Schütz et al., 2020). By comparing to these studies, it appears promising that Bro could be used as an anti-SARS-CoV-2 agent.

The ability of Bro to recognize and interact with RBD is evidenced by the number of interface residues and hydrogen bonds as presented in Table 3. The hydrogen bond has an important role in the stabilization of macromolecular interactions by helping to stabilize the three-dimensional structures of the protein (Gurusaran et al., 2016). The number of interface residues in the RBD variants is lower than in WT. The number of H-bonds is higher in Bro and RBD variants, although the number of bonds does not differ much among RBDs. Table 4 shows that Bro and RBDs exhibit numerous H-bonds and nonbonded interactions, indicating that Bro interacts well with RBDs. Nonbonded interactions are typically divided into two types: electrostatic and van der Waals interactions in force field representation (Duan et al., 2020). The salt bridges were contributed by Glu484 (E484) in RBD WT, United Kingdom, and US variants, where the mutation occurs, with Lys162 of Bro. Salt bridges are frequently associated with structural driving forces that increase the stability of the interaction (Pylaeva et al., 2018). The interface area indicates that the two groups of complexes of Bro and RBDs overlap in terms of affinities and buried surface areas, with no clear boundary separating them (Chen et al., 2013).

**TABLE 3:** Interface residues, area, and the bonds between Bro and RBDs.

RBD variants	No. of interface residues	Interface area (Å^2^)	No. of salt bridges	No. of disulphide bonds	No. of hydrogen bonds	No. of nonbonded contacts
WT	—	—	—	—	—	—
	26	1,199	2	-	13	162
	26	1,163				
SA	—	—	—	—	—	—
	29	1,173	1	-	14	170
	23	1,209				
United Kingdom	—	—	—	—	—	—
	29	1,176	2	-	15	178
	22	1,211				
BR	—	—	—	—	—	—
	29	1,172	1	-	13	171
	23	1,210				
United States	—	—	—	—	—	—
	28	1,199	3	-	17	220
	28	1,214				

**TABLE 4:** H-bonds between RBDs and Bromelain. Amino acids that are important in the interaction between RBD and hACE2 are marked with italics font, while bold font indicates the mutated amino acids.

RBD variants	RBD residues	Bromelain residues	H-bonds distance (Å)
Wild Type	Arg346	Ser115; Asp117	3.24; 2.75
	Tyr351	Tyr245	2.93
	Asp442	Gln338	2.95
	Lys444	Tyr232	2.52
	Asn448	Ser233; Tyr232; Thr336	2.79; 2.80; 2.88
	*Tyr449*	Gln338	3.05
	Glu484	Asp132; Lys162	2.93; 2.50
	*Gln493*	Val164	2.89
	*Gln498*	Ser339	2.96
SA	Arg346	Ser115; Asp117	2.78; 2.84
	Asn354	Asp117	3.24
	*Gly446*	Thr230	2.76
	Gly447	Thr336	2.77
	Asn448	Ser233	2.56; 2.80
	Asn450	Gln338; Asn249	3.07; 2.90
	Thr470; Glu471	Tyr133	3.01; 2.90
	*Gln493*	Val164	2.88
	Tyr495; *Gln498*	Gln338	2.89; 2.81
United Kingdom	Arg346	Ser115; Asp117	2.79; 2.82
	Asn354	Asp117	3.30
	*Gly446*	Thr230	2.75
	Gly447	Thr336	2.77
	Asn448	Ser333	2.57; 2.79
	Asn450	Gln338; Asn249	3.13; 2.91
	Thr470; Glu471	Tyr133	3.02; 2.90
	*Gln493*	Val164	2.89
	Phe495; Phe497; *Gln498*	Gln338	2.86; 3.25; 2.81
BR	Arg346	Ser115; Asp117	2.79; 2.85
	Asn354	Asp117	3.22
	*Gly446*	Thr230	2.76
	Gly447	Thr336	2.77
	Asn448	Ser333	2.55; 2.80
	Asn450	Gln338; Asn249	3.06; 2.90
	Thr470; Glu471	Tyr133	3.02; 2.90
	*Gln493*	Val164	2.86
	Tyr495; *Gln498*	Gln338	2.89; 2.81
United States	Thr345	Ser115	2.60; 2.60
	Arg346	Ser115; Asp117; Tyr245	2.68; 2.71; 2.75; 2.75
	Tyr351	Asn249	2.82
	Asn354	Asp117	2.81
	Lys356	Asp118	2.93
	Gly447	Thr230	2.81
	Asn448	Thr336	2.75
	Asn450	Asn249	2.81
	Ile468	Trp248	2.82
	Thr470	Tyr133	2.75
	Glu471	Tyr133	2.88
	Ser494	Ser339; Leu337	2.72; 2.95

### Molecular Dynamics Simulation Analysis

A molecular dynamics simulation was conducted to analyze the binding stability of Bro and RBD complexes, where multiple descriptors were analyzed to understand the flexible and stable nature of the complexes. The last frame interaction between Bro and RBDs in MD simulation is shown in Table 5. Between the two molecules, there was an interacting shift in the amino acid. However, interactions with amino acids continue to exist at the active sites of the RBDs. In RBD WT, Bro still interacted with Tyr449, Glu484, Gln493, Gly496, although the interacting amino acids of Bro were slightly different. The interaction of Bro with the RBD UK variant was quite stable with amino acids Tyr449, Gly446, Glu484, Gln493, Gly496, Gln498, and Tyr501, although the amino acids in the interacting Bro underwent slight changes. There was a slight shift in the interaction between Bro and the RBD BR variant, where the interaction only occurred at Tyr449, Lys484, Gln498, Thr500, and Tyr501. The Bro no longer interacted with Gly446, Gln493, and Gly496. Bro had another interaction, namely with Thr500. In the RBD SA variant, Bro interacted with Tyr449, Gly446, Lys484, Gly496, Gln498, and Tyr501, and no longer with Gln493. Meanwhile, in the final frame, Bro interacts more with the RBD US variant, specifically with residues Tyr449, Gly446, Glu484, Arg452, Gln493, Gly496, and Gly498, which did not initially interact with Gln493 and Gln498. Given this fact, the last frames of the Bro-RBD complex were reliable.

**TABLE 5:** Interaction between bromelain and RBDs of the last frame MD simulation on amino acids that are important in the interaction between RBD and hACE2 (marked with italics font). The bold font indicates mutated amino acids.

RBD variants	RBD	Bromelain	Distance (Å)		
	Residues	Residues	H-bonds	Salt-bridge	Nonbonded contacts
WT	*Tyr449*	Phe116	—	—	3.75
		Ser115	—	—	3.62
	Glu484	Asp132	2.73	—	3.88; 2.73; 3.61; 3.26
		Tyr133	—	—	3.76
		Gly134	—	—	3.58; 3.33; 3.89
		Lys162	2.90	2.90	3.51; 2.90; 3.72
	*Gln493*	Ile163	—	—	3.42; 3.81; 3.74; 3.88
		Val164	—	—	3.86
		Gly166	—	—	3.69
	*Gly496*	Ser339	—	—	3.88; 3.09; 3.76; 3.70; 3.84
	*Gln498*	Tyr228	2.79	—	3.74; 3.66; 3.85; 2.79
		Gln338	2.82	—	3.63; 3.21; 3.39; 2.82
		Ser339	—	—	3.65; 3.77
		Leu337	—	—	3.82
United Kingdom	*Tyr449*	Thr336	—	—	3.81; 3.60
		Tyr334	—	—	3.88; 3.89; 3.28
		Phe116	—	—	3.57; 3.65
	*Gly446*	Gln338	—	—	3.45; 3.61
	Glu484	Asp132	2.92	—	2.92; 3.64; 3.01
		Lys162	2.89	2.89	3.69; 3.36; 3.63; 2.89; 2.99; 3.09
		Tyr133	—	—	3.69; 3.84
		Gly134	—	—	3.81; 3.71
	*Gln493*	Ile163	—	—	3.23; 3.55; 3.67
		Val164	—	—	3.84; 3.25; 3.70; 3.09; 3.37; 3.74; 3.52
		Thr165	—	—	3.68; 3.74; 3.33; 3.67
		Gly166	—	—	3.58
	*Gly496*	Ser339	3.02	—	3.03
		Gln338	—	—	3.69; 3.88
	*Gln498*	Gln338	—	—	2.55; 3.77; 3.72; 3.80
	*Tyr501*	Ser339	—	—	3.78; 3.29; 3.54; 3.52; 3.11; 3.17; 2.95; 3.31
		Gln338	—	—	3.46; 3.83; 3.85
BR	*Tyr449*	Asp117	3.12	—	
		Asn120	2.73	—	3.70; 3.61; 3.77; 2.73
		Ser115	—	—	3.86
		Asp117	—	—	3.12; 3.76; 3.24; 3.23; 3.80; 3.38
	Lys484	Asp132	2.75	—	3.77; 2.75; 3.73; 379
		Tyr133	—	—	3.65; 3.65; 3.68; 3.18
	*Gln498*	Gln338	3.13	—	3.70; 3.61; 3.83; 3.46; 3.75; 3.13
		Thr336	—	—	3.36
	*Thr500*	Gln338	—	—	3.88
	*Tyr501*	Ser339	3.14	—	3.70; 3.57; 3.14; 3.20
			—	—	
SA	*Tyr449*	Ser115	2.85; 3.05	—	—
		Phe116	—	—	3.90
		Val114	—	—	3.72; 3.75; 3.84; 3.61; 3.69
		Ser115	—	—	3.39; 3.49; 3.56; 2.85; 3.68; 3.75; 3.05; 3.66
	*Gly446*	Gln338	2.69	—	3.48; 3.47; 3.51; 2.69
		Thr336	—	—	3.53; 3.69
	Lys484	Tyr133	—	—	3.58; 3.40; 3.86
	*Gly496*	Gln338	—	—	3.43; 3.39
	*Gln498*	Gln338	—	—	3.75; 3.68; 3.52; 3.59
		Thr230	—	—	3.68
	*Tyr501*	Ser339	2.75	—	3.04; 3.29; 3.39; 3.86; 3.68; 2.75; 3.18
		Gln338	—	—	3.56
United States	*Tyr449*	Thr336	—	—	3.60; 3.84
		Gln338	—	—	3.86; 3.12; 3.13; 3.07
	*Gly446*	Thr230	—	—	3.66; 3.40; 3.83; 3.75; 3.10; 2.68
		Gln338	—	—	3.85; 3.53
	Glu484	Asp132	2.69	—	3.89; 2.69; 3.71
		Tyr133	—	—	3.67; 3.29; 3.65; 3.45
		Lys162	3.01	2.90	3.44; 3.84; 3.01; 3.49; 3.36; 2.90
	Arg452	Asn249	—	—	3.84; 3.72; 2.84; 3.83; 3.81
		Gln250	—	—	3.89; 2.85
	*Gln493*	Val164	2.76	—	3.52; 3.54; 3.88; 3.42; 3.61; 3.50; 3.34; 3.80; 2.76
	*Gly496*	Ser339	—	—	3.66; 3.79; 3.21; 3.72; 3.72; 3.64
		Gln338	—	—	3.86
	*Gln498*	Gln338	3.01	—	3.01
		Thr230	—	—	3.84

**FIGURE 10:**
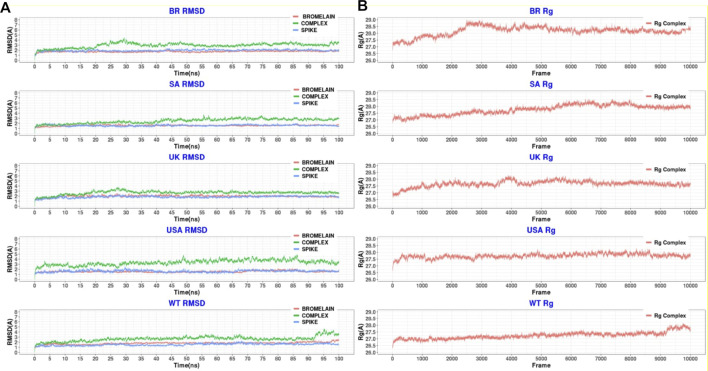
A molecular dynamics simulation. **(A)** Root mean square deviation of bromelain, RBDs, and the complex of bromelain and RBDs, and **(B)** Radius of gyration of the complex of bromelain and RBDs.

Figure 10 illustrates that bromelain-RBD complexes, RBD, and bromelain were relatively stable across the simulation times where lower flexibility was observed. The lower deviation in RMSD was not found in the complexes of the BR variant where high RMSD was observed after 20ns simulation time. This complex maintained a similar profile across the simulation trajectories, but it showed a comparatively less stable profile due to a higher trend in RMSD. The Bro-SA variant complex was relatively stable and exhibited lower RMSD. The complex followed a similar pattern, but after 40 ns, it began to rise slightly due to flexibility. However, the complex remained stable after that and maintained a similar pattern until the end of the simulations. The complex of the Bro-UK variant had lower RMSD than other variants, which defines a more rigid profile of the complex. However, the complex Bro-US variant had a comparatively large difference in RMSD. The RMSD value of Bro-WT was lower than other complexes, which indicates the stable profile of the complex.

Moreover, the radius of gyration of the simulated complexes was analyzed to understand the mobile nature of the complexes and the complexes’ flexibility (Figure 10). The higher Rg correlates with the higher level of labile nature of the complexes, whereas the lower Rg profile indicates the contracted nature of the complexes. The Bro-WT, United States, United Kingdom, and SA variant complexes had relatively stable and lower Rg compared to the Bro-BR variant complex. They maintained a steady-state throughout the whole simulation time.

The Bro-BR variant complex had flexibility after 20 ns time, and higher than the other variants, which indicates the labile nature of this complex. The bromelain-WT RBD complex stayed stable with a 2 Å RMSD value up to 10 ns, then the RMSD of complex slowly rose between 10–15 ns and reached 2.5 Å, and the RMSD value, which continued to increase slowly, was 3 Å at 25 ns and stabilization was seen at this value until 50 ns. Between 50–70 ns, the RMSD value fluctuated at around 3 Å after 70 ns, then at 92 ns, the complex was uninformed and maintained at the level of 2.5 Å. At 92 ns, the RMSD rose sharply to 4 Å with some ripple, and the RMSD reached 4.5 Å, but from 98 ns, the RMSD stabilized at 3.5 Å. The Rg graph was observed to support this result. The average Rg value for the complex during the simulation was calculated as 27.25 Å.

The Bro-US complex stayed stable with a 3 Å RMSD value up to 27 ns. Subsequently, the RMSD of the complex slowly rose between 27–85 ns and reached 4 Å, and slowly decreased to about 3 Å end of simulations. The Rg value of the complex was stable between 27 and 28 Å throughout 100 ns with an average value of 27.75 Å.

The Bro-UK complex stayed stable until the 8 ns of the long MD, while the RMSD value gradually increased until 3.5 Å among the 8–30 ns interval. Following that, the complex’s RMSD remained around 2.5 Å until the 100 ns long MD. The Rg value of the complex showed an upward trend from 26.75 to 28.25 Å during the first 40ns or 4k frames. Afterward, in the early 40 ns, the Rg value decreased and stayed steady at around 27.5 Å until the end of 100 ns The average Rg value for the simulation was determined as 27.66 Å.

The Bro-SA complex was stable until 40 ns. After that, the RMSD value rose to 3 Å and even with some fluctuations, the RMSD value was stable at around 3 Å until the 100 ns The Rg graph of the complex showed an upward trend from 27.00 to 28.5 Å throughout the first 65 ns From 65 to 85 ns, some waves were observed in the Rg value, but after 85 ns until 100 ns, the value was stable, where the Rg value was observed at around 28.00 Å. The average value was observed as 27.76 Å.

The Bro-BR complex was stable until the 20 ns at below 2 Å, and then increased to about 4 Å up to 30 ns, and was stable with a small deviation between 35 and 100 ns The complex Rog is similar to the RMSD profile, and it reached 29 Å value until 30 ns and was stable at about 28.5 Å after 40 ns Lastly, the average Rg value of the complex was calculated as 28.16 Å.

**FIGURE 11:**
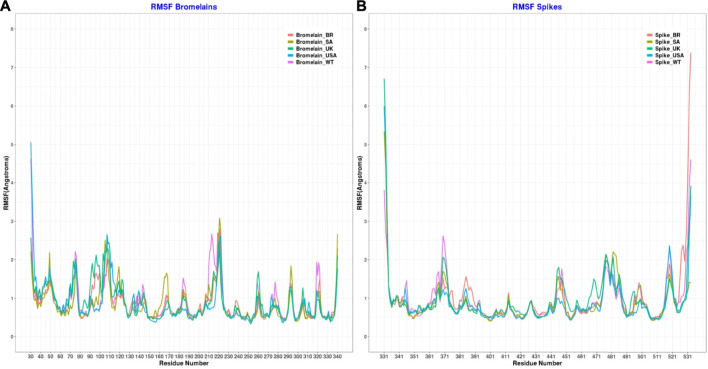
Root mean square fluctuation of **(A)** the complex of bromelain and RBDs, and **(B)** the RBDs.

Moreover, the root mean square fluctuations (RMSFs) of the simulated complexes were analyzed to understand the flexibility across the amino acid residues of the complexes Figure 11). The RMSF profile in their amino acid residues had lower RMSF than 2.5 Å, except for the BR variant on Asn221, SA variant on Asn107, Ala220, Asn221, Ser222, non on United Kingdom variant, United States variant on Asp31, Pro32, Asn107, Ser222, and WT variant on Asp31, Ala213, Ala219, Ala22. Moreover, the RBD had a similar lower RMSF profile for maximum residues, except for BR variant on Ile332, Thr333, Ser530, Thr531, Asn532, SA on variant Ile332, United Kingdom variant on Ile332, Thr333, Asn532, United States variant on Ile332, Thr333, Asn532, and WT on Ile332, Asn370, Asn532, which defines the stable and lower flexibility of the complex. However, there exceptions. In all RBD variants, regions 366–372, 445–448, 475–486, and 517–521 are relatively more flexible than the rest of the protein with RMSF values between 1.5–2.5Å.

Moreover, binding free energy (BFE) calculations were also performed for the 100 ns long MDs with 10,000 frames, with the 50 frames interval of each complex. Calculations were done as explained in the Methods part. Respectively, the complexes binding free-energy (∆G) were calculated as −125.89 kcal/mol (WT), −74.24 (BR variant), −80.80 (SA variant), −98.47 (United Kingdom variant) and −106.6 (US variant) (Table 6). Binding analyses revealed differences in the interactions of Bro and WT, BR variant, SA variant, United Kingdom variant, and US variant, with Bro’s binding affinity being stronger on WT than its variants. The complex Bro-BR variant has the least interaction.

**TABLE 6:** Free binding energy (ΔG MM-GBSA) of bromelain and RBD complexes.

RBD variants	Van der Waals energy	Electrostatic energy	Polar solvation energy	Sasa energy	ΔG MM-gbsa (kcal/mol)
WT	−124.19 ± 0.70	−759.04 ± 4.17	774.36 ± 3.68	−17.02 ± 0.09	−125.89 ± 1.12
BR	−95.62 ± 1.16	−855.16 ± 3.19	889.61 ± 3.11	−13.08 ± 0.14	−74.24 ± 1.15
SA	−102.38 ± 1.09	−855.10 ± 3.40	890.34 ± 3.34	−13.65 ± 0.13	−80.80 ± 1.21
United Kingdom	−110.97 ± 0.96	−704.42 ± 3.40	732.03 ± 3.73	−15.12 ± 0.13	−98.47 ± 1.32
United States	−113.27 ± 0.72	−916.50 ± 2.78	938.67 ± 2.52	−15.55 ± 0.10	−106.65 ± 0.84

## Conclusion

The interaction between bromelain and various RBD variants of SARS-CoV2 was reported for the first time in this study. The detailed in silico analysis suggests that bromelain effectively binds to RBD’s active site, especially with the residues Gln493, Tyr449, Gly446, Gly496, and Gln498. As a result, the interaction between RBD and hACE2 was hypothetically interfered with significantly, thus suggesting the potential use of bromelain to prevent viral entry into the host cells. The current study could lay the groundwork for bromelain to be used as an inhibitor against SARS-CoV-2, but more *in vitro* and *in vivo* testing is needed before making any final conclusion.

## Data Availability

The raw data supporting the conclusions of this article will be made available by the authors, without undue reservation.
